# Trabeculectomy followed by phacoemulsification versus trabeculectomy alone: The Collaborative Bleb-Related Infection Incidence and Treatment Study

**DOI:** 10.1371/journal.pone.0223439

**Published:** 2019-10-24

**Authors:** Shogo Arimura, Kentaro Iwasaki, Makoto Gozawa, Yoshihiro Takamura, Masaru Inatani

**Affiliations:** Department of Ophthalmology, Faculty of Medical Science, University of Fukui, Yoshida, Fukui, Japan; Massachusetts Eye & Ear Infirmary, Harvard Medical School, UNITED STATES

## Abstract

**Purpose:**

This study aims to compare the rate of surgical failure after trabeculectomy followed by phacoemulsification vs trabeculectomy alone for 5 years.

**Method:**

A total of 1,098 eyes of patients with glaucoma who underwent trabeculectomy with mitomycin C at 34 clinical centers included in CBIITS were analyzed. During follow-up, some eyes were treated with phacoemulsification because of cataract progression. The patients were divided into the “trabeculectomy followed by phacoemulsification” and “trabeculectomy alone” groups, and surgical probabilities were compared. Surgical failure was defined on the basis of mean IOP as follows; < 20% reduction in preoperative IOP or IOP ≥ 21 mmHg (criterion A), IOP ≥ 18 mmHg (criterion B), or IOP ≥ 15 mmHg (criterion C).

**Result:**

In total, 40 eyes were treated with trabeculectomy followed by phacoemulsification and 208 with trabeculectomy alone. Preoperative intraocular pressure was 22.1 ± 8.7 mmHg in the trabeculectomy followed by phacoemulsification group and 20.5 ± 6.3 mmHg in trabeculectomy alone group (*P* = 0.47). The 5-year cumulative probabilities of success in the trabeculectomy followed by phacoemulsification and trabeculectomy alone groups were respectively 40.0% and 59.1% for criterion A (*P* = 0.01), 35.0% and 52.9% for criterion B (*P* = 0.01), and 25.0% and 37.5% for criterion C (*P* = 0.08). Cox proportional hazards regression model indicated that shorter time gap between trabeculectomy and phacoemulsification was associated with surgical failure.

**Conclusion:**

Phacoemulsification following trabeculectomy adversely affects surgical outcomes. In particular, a shorter time gap between trabeculectomy and phacoemulsification reduces the probability of success.

## Introduction

Trabeculectomy is a common filtering surgery for patients with glaucoma with medically uncontrollable intraocular pressure (IOP); however, cataract often progresses after trabeculectomy [1–5]. When cataract surgery is considered for such patients, early increases in IOP and long-term control of IOP are of great concern. Effects of cataract surgery on IOP control in eyes that have undergone trabeculectomy are controversial. Several reports [6–8] have demonstrated that phacoemulsification following trabeculectomy reduces bleb formation, results in increased IOP, and requires additional medications to control IOP. Meanwhile, some other studies [9–11] have shown no adverse effects on IOP or the number of glaucoma medications.

The Collaborative Bleb-Related Infection Incidence and Treatment Study (CBIITS) [12] was a prospective multicenter study including 1,249 eyes. CBIITS investigated the incidence, severity, and prognosis of bleb-related infections following trabeculectomy with mitomycin C (MMC) for 5 years. Currently, few studies conducted in large cohorts and over long periods have assessed effects of phacoemulsification following trabeculectomy [13, 14]. Therefore, the present study aimed to compare the long-term outcomes in terms of resultant IOP between trabeculectomy followed by phacoemulsification and trabeculectomy alone and to identify the factors affecting IOP changes based on CBIITS data.

## Materials and methods

### Patient selection

The CBIITS protocol has been described in detail elsewhere [12]. All institutions participating in the study obtained approval from the relevant medical center. Written informed consent was obtained from all patients, and the protocol conformed to the tenets of the Declaration of Helsinki. Briefly, patients were enrolled for 2 years and followed up every 6 months for up to 5 years. The enrolled patients were treated with filtrating surgery. Ophthalmological examinations were completed at each follow-up visit according to the protocol. CBIITS enrolled 1,249 cases (1,249 eyes) for 2 years, and enrollment was completed on March 31, 2007. The study finally included 1,098 eyes treated with trabeculectomy and 0.04% MMC.

The present study was approved by the institutional review board of Fukui University Hospital, Fukui, Japan. Patients from CBIITS database who met the following criteria were recruited: (1) Japanese patients with primary open-angle or exfoliation glaucoma, (2) those with a minimum age of 20 years, (3) those treated with trabeculectomy with 0.04% MMC, and (4) those with phakic eyes. Patients were excluded based on the following criteria: (1) those with any previous surgeries for glaucoma, (2) those with previous vitreoretinal surgeries, (3) trabeculectomy combined with lens extraction, or (4) those with any postoperative intraocular surgeries except for glaucoma reoperation or phacoemulsification.

The enrolled patients were divided into 2 groups: trabeculectomy followed by phacoemulsification and trabeculectomy alone. The trabeculectomy followed by phacoemulsification group comprised eyes that had undergone lens extraction with phacoemulsification during the 5-year follow-up period. The trabeculectomy alone group comprised eyes without postoperative lens extraction during the 5-year follow-up period. In CBIITS, indication for surgery, selection of operative procedure, operative technique, and application of postoperative medication or additional glaucoma treatment, either medically or surgically, was at the discretion of local investigators. Trabeculectomy was performed with 0.04% MMC, and fornix-based or limbus based-conjunctival incision. Cataract surgery was performed with clear corneal incision or superior conjunctival incision, excluding extracapsular cataract extraction.

### Primary outcome

The primary outcome was surgical failure, as determined based on IOP. In CBIITS, IOP was recorded post trabeculectomy at each clinical center, and the mean IOP every 6 months was conveyed to the CBIITS data analysis center at Gifu University in Japan. In the present study, the cumulative probabilities of surgical failure based on CBIITS data were compared between the 2 groups. Surgical failure was defined on the basis of mean IOP at every 6-month interval after trabeculectomy with or without antiglaucoma medications: <20% reduction in preoperative IOP or IOP ≥ 21 mmHg (criterion A), IOP ≥ 18 mmHg (criterion B), or IOP ≥ 15 mmHg (criterion C). Additionally, surgical failure was declared in cases that required reoperation for glaucoma or developed loss of light perception or low IOP (≤5 mmHg). Reoperation for glaucoma was defined as the requirement of bleb needling after >6 months of trabeculectomy, bleb revision, or additional glaucoma surgery. Laser suture lysis or bleb needling within 6 months following trabeculectomy was not considered as surgical failure because it was a part of the postoperative management for trabeculectomy.

### Secondary outcome

The secondary outcome was presence of prognostic factors for surgical failure on the basis of IOP in each criterion (A, B, and C).

### Sample size

In a previous report^7^ about IOP change after phacoemulsification in eyes with previous trabeculectomy, IOP reduction appeared to be the same or superior in trabeculectomy alone group. Therefore, we performed one-sided p value for the estimation of the sample size to show that trabeculectomy alone group has superiority to trabeculectomy followed by phacoemulsification about postoperative IOP reduction. If difference in surgical failure rate between the 2 groups was ≥20% with a one-sided significance level of 0.05 and a power of 0.8, an estimated sample size of at least 186 eyes was calculated as essential to detect a significant difference between the 2 groups.

### Statistical analysis

JMP package version 10.0 (SAS Institute, Inc. Cary, NC, USA) was used for statistical analysis. The normality of the data was checked with Shapiro-Wilk test. Univariate analyses were performed using Wilcoxon non-parametrical and chi-square tests. The 5-year cumulative probabilities of surgical failure were analyzed using Kaplan–Meier survival curves and log-rank test. Multivariate analysis was performed using Cox proportional hazards regression model to determine prognostic factors for surgical failure. We performed the likelihood-ratio test to check the validity of Cox proportional hazards regression analysis. P values were respectively < 0.01 (criterion A), 0.02 (criterion B) and 0.08 (criterion C).

## Results

### Patient recruitment

Table 1 summarizes the patient characteristics. There were 40 and 208 eyes that were treated with trabeculectomy followed by phacoemulsification and trabeculectomy alone, respectively. There were significant differences in patient age (*P* < 0.01) and the number of preoperative glaucoma medications (*P* = 0.02) between the 2 groups. Preoperative IOP (mean ± SD) was not significantly different between the trabeculectomy followed by phacoemulsification (22.1 ± 8.7 mmHg) and trabeculectomy only (20.5 ± 6.3 mmHg; *P* = 0.47) groups. The median time gap between trabeculectomy and phacoemulsification was 24 months.

**Table 1:** Patient characteristics.

Factor	Trabeculectomy Followed by Phacoemulsification Group	Trabeculectomy Alone Group	*P* value
	n = 40	n = 208	
**Age (years)**	67.9 ± 9.0	59.9 ± 11.2	<0.01*
**Sex (female/male)**	20/20	77/131	0.13
**Type of glaucoma (XFG/POAG)**	8/32	22/185	0.12
**LogMAR BCVA**	0.12 ± 0.24	0.16 ± 0.43	0.14
**IOP (mm Hg)**	22.1 ± 8.7	20.5 ± 6.3	0.47
**Number of glaucoma medications (n)**	2.3 ± 1.2	2.8 ± 1.0	0.02*
**Conjunctival incision (Fornix-based/limbus-based)**	15/25	69/139	0.60
**Refractive value (D)**	−2.6 ± 4.1	−3.3 ± 4.1	0.20
**MD value (dB)**	−18.4 ± 7.2	−18.1 ± 8.6	0.90

Data are presented as mean ± standard deviation

XFG = exfoliative glaucoma, POAG = primary open-angle glaucoma, LogMAR = logarithm of the minimal angle of resolution, BCVA = best collective visual acuity IOP = intraocular pressure, D = diopter, MD = mean deviation

*Wilcoxon’s nonparametric test.

**Fig 1:**
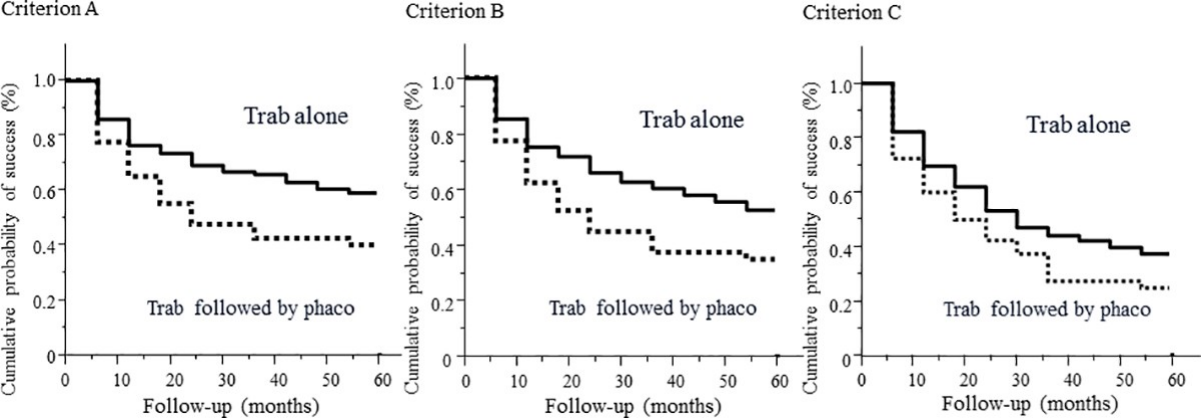
Kaplan–Meier survival curves of the 5-year probability of success in the trabeculectomy followed by phacoemulsification and trabeculectomy alone groups. (Left) For criterion A (intraocular pressure ≥ 21 mm Hg; <20% reduction of the preoperative intraocular pressure), (Center) for criterion B (intraocular pressure ≥ 18 mm Hg; <20% reduction of the preoperative intraocular pressure), and (Right) for criterion C (intraocular pressure ≥ 15 mm Hg; <20% reduction of the preoperative intraocular pressure). Surgical failure in all the criteria includes reoperation for glaucoma, loss of light perception, or low intraocular pressure (≤5 mm Hg). Trab = Trabeculectomy, Phaco = Phacoemulsification.

The 5-year cumulative probabilities of success in the trabeculectomy followed by phacoemulsification and trabeculectomy alone groups were 40.0% and 59.1% for criterion A (*P* = 0.01), 35.0% and 52.9% for criterion B (*P* = 0.01), and 25.0% and 37.5% for criterion C (*P* = 0.08), respectively (Fig 1). Five eyes (12.5%) in the trabeculectomy followed by phacoemulsification group were subjected to phacoemulsification within 1 year after trabeculectomy, and surgical failure on the basis of all criteria was observed in 4 of the 5 eyes. Failure due to insufficient IOP reduction was observed in 17 (43%) eyes based on criterion A, 19 (48%) eyes based on criterion B, and 23 (58%) eyes based on criterion C in the trabeculectomy followed by phacoemulsification group, and insufficient IOP reduction was observed in 59 (28%) eyes based on criterion A, 72 (35%) eyes based on criterion B, and 104 (50%) eyes based on criterion C in the trabeculectomy alone group (Table 2).

**Table 2:** Insufficient IOP reduction for surgical failure.

Criterion	Trabeculectomy Followed by Phacoemulsification Group	Trabeculectomy Alone Group	*P* value
	n = 40	n = 208	
**A**	n = 17 (43%)	n = 59 (28%)	0.10
**B**	n = 19 (48%)	n = 72 (35%)	0.13
**C**	n = 23 (58%)	n = 104 (50%)	0.42

IOP = intraocular pressure

The data were analyzed with chi-square test.

Surgical failure due to the loss of light perception was not observed in any patient who completed the 5-year follow-up. Bleb needling after 6 months or reoperation was required in 3 (8%) eyes in the trabeculectomy followed by phacoemulsification group and in 12 (6%) eyes in the trabeculectomy alone group. Low IOP was found in 4 eyes (10%) in the trabeculectomy followed by phacoemulsification group and 14 eyes (7%) in the trabeculectomy alone group (Table 3).

**Table 3:** The reason for surgical failure.

Factor	Trabeculectomy Followed by Phacoemulsification Group	Trabeculectomy Alone Group	*P* value
	n = 40	n = 208	
Loss of light perception	n = 0 (0%)	n = 0 (0%)	
Bleb needling after 6 months or reoperation	n = 3 (8%)	n = 12 (6%)	0.67
Low IOP	n = 4 (7%)	n = 14 (10%)	0.47

IOP = intraocular pressure

Surgical failure due to the loss of the light perception was not observed in all the patients.

The data were analyzed with chi-square test.

### Prognostic factors for trabeculectomy failure

Baseline characteristics, including age, glaucoma type, preoperative logMAR best collective visual acuity, preoperative IOP, number of glaucoma medications, type of conjunctival flap incision (fornix-based or limbus-based), mean deviation, and time gap between trabeculectomy and phacoemulsification were evaluated as prognostic factors for surgical failure (Table 4). Three factors were significant in the Cox proportional hazards regression models. Shorter time gap between trabeculectomy and phacoemulsification was significantly associated with surgical failure in criteria A [hazard ratio (HR) = 0.98; *P* = 0.01], B (HR = 0.98; *P* < 0.01), and C (HR = 0.98; *P* = 0.03). Lower preoperative IOP was significantly associated with surgical failure in criteria A (HR = 0.94; *P* < 0.01) and B (HR = 0.97; *P* = 0.01). Higher logMAR values of the preoperative best collective visual acuity were significantly associated with surgical failure in criteria A (HR = 2.14; *P* < 0.01), B (HR = 1.96; *P* < 0.01), and C (HR = 1.93; *P* < 0.01).

**Table 4:** Hazard ratio analyzed using multivariate cox proportional hazards regression models.

Factor	Criterion					
	A		B		C	
	HR (95% Cl)	*P* value	HR (95% Cl)	P value	HR (95% Cl)	*P* value
**Age (years)**	0.99 (0.98–1.01)	0.24	0.99 (0.98–1.01)	0.25	0.99 (0.98–1.01)	0.38
**Type of glaucoma (XFG/POAG)**	1.12 (0.66–1.83)	0.66	0.92 (0.53–1.52)	0.75	0.79 (0.45–1.34)	0.40
**Preoperative logMAR BCVA per 1.0**	2.14 (1.32–3.30)	< 0.01*	1.96 (1.21–3.02)	< 0.01*	1.93 (1.21–2.95)	< 0.01*
**Preoperative IOP per mmHg**	0.94 (0.92–0.98)	< 0.01*	0.97 (0.94–0.99)	0.01*	0.99 (0.96–1.01)	0.33
**The number of preoperative glaucoma medication per each**	0.98 (0.86–1.13)	0.81	0.97 (0.85–1.11)	0.68	0.99 (0.86–1.13)	0.83
**Conjunctival incision (Fornix-based/limbus-based)**	0.90 (0.64–1.27)	0.56	0.84 (0.59–1.18)	0.31	0.84 (0.59–1.18)	0.31
**MD value per dB**	1.01 (0.99–1.03)	0.34	1.01 (0.99–1.02)	0.23	1.01 (0.99–1.03)	0.18
**Period between trabeculectomy and phacoemulsification per month**	0.98 (0.96–0.99)	0.01*	0.98 (0.96–0.99)	<0.01*	0.98 (0.97–0.99)	0.03*

XFG = exfoliative glaucoma, POAG = primary open-angle glaucoma, LogMAR = logarithm of the minimal angle of resolution, BCVA = best collective visual acuity, IOP = intraocular pressure, MD = mean deviation, HR = hazard ratio

*P < 0.05

## Discussion

The present study compared the outcomes of IOP between trabeculectomy followed by phacoemulsification and trabeculectomy alone over 5 years. The rate of surgical failure was higher in the trabeculectomy followed by phacoemulsification group than in the trabeculectomy alone group based on criteria A and B. Multivariate analysis using Cox proportional hazards regression model revealed that a shorter time gap between trabeculectomy and phacoemulsification, lower preoperative IOP, and higher preoperative logMAR BCVA value were significantly associated with surgical failure.

Many reports have evaluated IOP control with phacoemulsification in eyes following trabeculectomy; however, only 2 studies [13,14] have assessed the risk associated with phacoemulsification following trabeculectomy over a long-term follow-up period (>2 years). In one study [13], 235 patients with glaucoma who had undergone cataract surgery after trabeculectomy (including extracapsular cataract extraction) or trabeculectomy alone with or without 5-fluorouracil were prospectively assessed. In another study [14], outcomes of patients with primary open-angle glaucoma who underwent trabeculectomy with 0.04% MMC alone (n = 108) or trabeculectomy with 0.04% MMC followed by cataract surgery (n = 108) were compared over a long follow-up period (mean follow-up period, 66 months).

Our findings are consistent with those of several previous studies assessing effects of phacoemulsification on postoperative IOP in eyes treated with trabeculectomy [14–18]. Bleb dysfunction has been hypothesized to be related to bleb fibrosis caused by upregulated cytokines in the anterior chamber following phacoemulsification [19–22]. Kawai et al. [23] and Inoue et al. [24] have demonstrated that the fibrogenic cytokine monocyte chemotactic protein-1 (MCP-1) was elevated in pseudophakic eyes. Aqueous MCP-1 levels are associated with trabeculectomy failure [25]. Siriwardena et al. [26] compared anterior chamber flare levels between patients who underwent trabeculectomy or phacoemulsification and found that postoperative flare levels in patients who underwent phacoemulsification were significantly higher than those in patients who underwent trabeculectomy after 3 months of surgery. Compared with baseline levels, the flare levels remained elevated until 6 months after phacoemulsification. In contrast, flare levels in patients who underwent trabeculectomy returned to normal in 4 weeks after surgery. The prolonged elevated flare levels in the anterior chamber might be related to lens crystalline material, ultrasound energy, or fluid irrigation. The elevated flare levels might be related to high MCP-1 levels in the aqueous humor.

A previous study [14] has demonstrated that surgical failure is frequent if the time gap between trabeculectomy and lens extraction is short. The concern is the optimal time for phacoemulsification to reduce the risk of trabeculectomy failure. Awai-Kasaoka et al. [27] found that the risk of failure significantly increased when phacoemulsification was performed within 1 year after trabeculectomy. In the present study, 5 eyes (12.5%) were subjected to phacoemulsification within 1 year after trabeculectomy, and surgical failure on the basis of all criteria was observed in 4 of the 5 eyes. Bleb remodeling lasts for at least 6 months after surgery [28,29]; thus, premature timing of phacoemulsification before the completion of bleb remodeling might accelerate bleb fibrosis.

Multivariate analysis using Cox proportional hazards regression model identified 2 additional prognostic factors for surgical failure: lower preoperative IOPs in criteria A and B and worse BCVA in all criteria. Lower preoperative IOPs may be a common prognostic factor [30]. In addition to the greater risk for hypotony, achieving ≥20% reduction in IOP is more difficult in eyes with lower preoperative IOPs than in those with higher preoperative IOPs. The prognostic factors might be related to the definition of surgical failure. Worse BCVA might reflect more severe glaucoma, which may thereby lead to worse surgical prognosis after trabeculectomy. Another explanation is that worse BCVA is related to advanced stage cataract requiring greater ultrasound energy during phacoemulsification. Further surgical invasion might result in subsequent bleb failure.

This study has some limitations. The patients were not randomly assigned to the groups. The 2 groups were significantly different in terms of age and received different numbers of glaucoma medications at the baseline measurement. The overall validity of Cox proportional hazards regression model for criterion C was not significant (*P* = 0.08). Therefore, the analysis for criterion C may not be guaranteed precisely. The surgical techniques for trabeculectomy used by the study centers or surgeons were not identical. To reduce these inherent biases, we need to accumulate well-designed cohort studies for good quality meta-analysis in the future.

## Conclusions

Additional phacoemulsification deteriorates the outcome of trabeculectomy in patients with primary open-angle glaucoma and exfoliation glaucoma. A longer time gap between trabeculectomy and phacoemulsification leads to better outcomes of trabeculectomy.

## Supporting information

S1 File Analysis datasets. (XLSX)
